# Promising Pre-Lithiation Agent Li_2_C_2_O_4_@KB for High-Performance NCM622 Cell

**DOI:** 10.3390/ma18194467

**Published:** 2025-09-25

**Authors:** Boqun Xia, Guangwan Zhang, Feng Tao, Meng Huang

**Affiliations:** 1 Sanya Science and Education Innovation Park, Wuhan University of Technology, Sanya 572000, China; 13407158120@163.com (B.X.); guangwanzhang@whut.edu.cn (G.Z.); t18438610309@163.com (F.T.); 2 State Key Laboratory of Advanced Technology for Materials Synthesis and Processing, School of Materials Science and Engineering, Wuhan University of Technology, Wuhan 430070, China

**Keywords:** lithium-ion batteries, cathode lithium supplementation, Li_2_C_2_O_4_, high energy density, spray drying

## Abstract

In conventional lithium-ion batteries (LIBs), active lithium loss during solid electrolyte interphase (SEI) formation reduces coulombic efficiency and energy density. Cathode pre-lithiation can effectively compensate for this irreversible lithium consumption. To address limitations of conventional pre-lithiation agents—such as complex synthesis and air instability—a Ketjen black-coated lithium oxalate nanocomposite (Li_2_C_2_O_4_@KB) using high-energy ball milling and spray drying was developed. This composite leverages the advantages of Li_2_C_2_O_4_, including a mild decomposition potential (4.26 V vs. Li^+^/Li), high theoretical lithium compensation capacity (525 mAh·g^−1^), and environmentally benign decomposition products, and significantly improves electronic conductivity and reduces particle size. When incorporated in NCM622 full cells, the initial capacity is increased by 18.21 mAh·g^−1^ at 0.3 C, with a 29.22% enhancement in capacity retention after 50 cycles at 0.3 C. At 1 C, the initial capacity is higher by 15.79 mAh·g^−1^, accompanied with a 7.72% improvement in retention after 100 cycles. The Li_2_C_2_O_4_@KB composite exhibits great promise as a practical and efficient cathode pre-lithiation additive for next-generation high-energy-density LIBs.

## 1. Introduction

The escalating environmental pollution crisis, primarily driven by persistent fossil fuel consumption, has intensified the global imperative to transition toward sustainable energy sources [1]. In response, advanced energy storage systems—especially electrochemical batteries—have gained prominence as key technologies for grid energy storage. Major categories include metal–air, lithium–sulfur, flow, and metal-ion batteries [2,3,4]. Metal–air batteries, employing lithium (5928 Wh kg^−1^), zinc (1218 Wh kg^−1^), magnesium (4032 Wh kg^−1^), aluminum (4332 Wh kg^−1^), and iron (763 Wh kg^−1^) anodes, represent promising high-energy-density systems for next-generation applications [2]. However, they often suffer from limited cycle life and environmental sensitivity. Lithium–sulfur (Li–S) batteries, with a theoretical energy density of ~2600 Wh kg^−1^, exceed conventional lithium-ion systems and rival lithium–air batteries [3]. They employ sulfur cathodes and lithium metal anodes, offering high specific capacity, environmental compatibility, and low cost. Yet, practical deployment is hindered by the polysulfide shuttle effect, sulfur cathode volume expansion, and lithium anode instability, leading to reduced cycle life and Coulombic efficiency. Flow batteries, including vanadium (25–50 Wh L^−1^) and zinc–bromine (50–75 Wh L^−1^) systems, are well-suited for large-scale, long-duration storage and renewable integration [4]. Although they exhibit high scalability and long cycle life, their energy density remains relatively low and upfront costs significant. Metal-ion batteries, particularly lithium-ion batteries (LIBs), have high energy density and excellent cycle life, establishing them as dominant solutions in current energy storage markets [5,6].

However, a significant challenge in current LIBs is the irreversible consumption of active Li^+^. These Li^+^, originating from lithium-containing oxide or phosphate cathodes, are depleted during the formation of the solid electrolyte interphase (SEI) on the anode surface, a process that is accompanied by the decomposition of the liquid electrolyte [7,8,9]. This results in a coulombic efficiency below 100% (e.g., 90–95% in the first cycle), significantly reducing the energy density and cycle life of LIBs. Next-generation high-energy-density LIBs employing high-capacity anodes—such as silicon (Si), tin (Sn), and phosphorus (P), which operate via alloying mechanisms—undergo significantly more severe side reactions compared to conventional graphite anodes. This heightened-capacities result in substantially greater initial lithium loss, often exceeding 15%, due to extensive SEI formation and irreversible consumption of Li^+^ during the first cycle [10,11]. Furthermore, due to large volumetric changes in these materials during lithiation (Si ≈ 420%, Sn ≈ 260%, P ≈ 300%), significant Li^+^ loss occurs. Side reactions in these high-capacity anodes persist for several or even dozens of cycles before Coulombic efficiency stabilizes above 99.9% [12,13]. Consequently, this irreversible Li^+^ loss substantially diminishes the available lithium inventory, leading to a marked reduction in the practical capacity and overall energy density of the battery.

Pre-lithiation has been extensively investigated as a highly promising approach to compensate for initial lithium loss and thereby improve the overall energy density of LIBs. Pre-lithiation is a technique designed to introduce supplemental active lithium into the battery prior to electrochemical cycling. This can be achieved through the use of specific chemical reagents, functional materials, or engineered processes. It is important to note that pre-lithiation is conceptually distinct from the physical or chemical pretreatment of electrode materials, representing an independent strategy for initial lithium compensation [14,15,16]. Anode pre-lithiation is commonly accomplished through the direct introduction of supplemental lithium sources, such as lithium powder, thin lithium foil, or specific lithium-containing compounds, into the anode structure prior to or during cell assembly [17,18,19]. However, the high reactivity of these materials and the stringent environmental conditions required for their handling cast doubt on their practical feasibility for large-scale commercial applications.

In contrast, cathode lithium supplementation shows more promise, as it enables direct addition of lithium supplementation materials to the slurry, highly compatible with established LIBs manufacturing processes [20,21,22,23,24]. Typically, cathode pre-lithiation employs Li-rich materials such as Li_2_Ni_0.5_Mn_1.5_O_2_, Li_1+x_Ni_0.65_Mn_0.2_Co_0.15_O_2_, Li_2_NiO_2_, Li_5_FeO_4_, and Li_6_CoO_4_, which are compatible highly compatible with established lithium-ion battery manufacturing processes [25,26,27]. However, their lithium supplementation capacity and stability are often inadequate. Additionally, their decomposition products are inert transition metal oxides, and the residues degrade the capacity of LIBs. Recently, gas-releasing pre-lithiation reagents, including Li_2_O and Li_3_N, have emerged as promising candidates owing to their high lithium donation capacity, which—following gas evolution—is comparable to that of metallic lithium. However, they have a very high electrochemical activity as well as a very high price, which is not conducive to the industrialization. From a commercialization perspective, low-cost and air-stable organic lithium salts with non-toxic byproducts, high lithium supplementation capacity and low operating voltage are considered excellent candidates as lithium supplementation materials [28,29].

**Figure 1:**
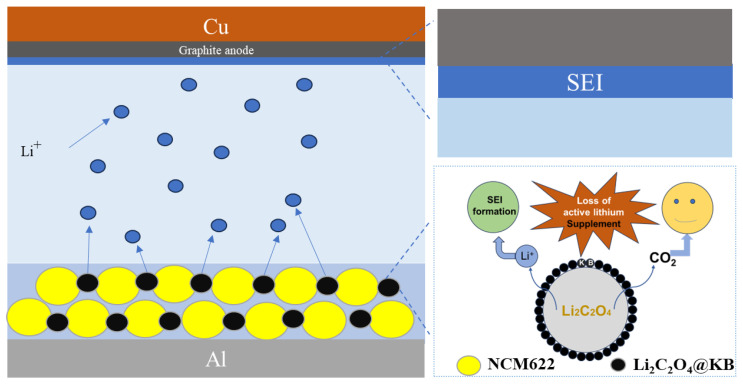
Lithium-ion compensation process and mechanism of Li_2_C_2_O_4_@KB composite.

Currently, the most widely utilized lithium supplementation agents include Li_5_FeO_4_, Li_2_NiO_2_, Li_6_CoO_4_, Li_2_S, Co/Li_2_O, and Co/LiF [9,30,31,32], and Table 1 summarizes the theoretical specific capacities of the Li_2_C_2_O_4_ and others. It can be observed that only Li_5_FeO_4_ and Li_2_S exhibit higher theoretical specific capacities than Li_2_C_2_O_4_. However, Li_5_FeO_4_ is unstable in the air and its decomposition generates excessive metal oxide deposits, which can diminish the overall energy density of the battery. Meanwhile, Li_2_S is not only costly but also produces sulfide-based by-products that pose considerable environmental concerns. In contrast, the low-cost Li_2_C_2_O_4_ offers superior environmental stability and well-balanced overall performance, making it a more sustainable and practically viable option. So, we selected lithium oxalate (Li_2_C_2_O_4_) as the pre-lithiation agent due to its high specific capacity (525 mAh g^−1^), low cost, environmental compatibility, and excellent stability in ambient air [33,34]. As illustrated in Figure 1, the Li_2_C_2_O_4_@KB composite releases extra Li^+^ to compensate for the irreversible consumption of lithium resulting from SEI formation during the initial charging, thereby enhancing the CE of the LIBs. To reduce the decomposition voltage of Li_2_C_2_O_4_, the predominant strategy involves the incorporation of catalytic substances, such as nickel (Ni) and manganese (Mn). These catalysts have been demonstrated to effectively facilitate the decomposition reaction at significantly lower potentials [35,36]. However, due to the complexity of these methods, it is difficult to prepare them on a large scale. We proposed a new method via encapsulating Li_2_C_2_O_4_ with Ketjen black, aiming to effectively reduce the decomposition voltage of Li_2_C_2_O_4_. In comparison to existing methodologies, the proposed strategy not only offers significantly improved process convenience, but also demonstrates enhanced scalability for industrial production, thereby better aligning with practical manufacturing requirements. The core–shell structured Li_2_C_2_O_4_@KB nanocomposite shows a decomposition potential below 4.26 V and provides a lithium supplementation capacity of 476 mAh g^−1^.

## 2. Materials and Methods

### 2.1. Synthesis of the Cathode Lithium Replenishment Material

Lithium formate (Li_2_C_2_O_4_) (Canrd, Dongguan, China) and KB (Canrd, Dongguan, China) were thoroughly mixed and ball-milled. Typically, 1 g of Li_2_C_2_O_4_ and 0.5 g of KB are added to the stainless-steel ball milling jar. The dispersion medium is anhydrous ethanol. Small (ø 1 mm) and large (ø 3 mm) zirconia balls are used in a 3:1 mass ratio and milled in the ball mill at 500 rpm for 6 h. After milling, the mixture was dried at 80 °C for 12 h to obtain the intermediate powder. This powder was then dispersed in 50 mL of deionized water, subjected to ultrasonication for 1 h, and subsequently stirred magnetically for 6 h. The resulting suspension was finally spray-dried at 180 °C with a liquid feed rate of 1 L·h^−1^ to produce the final Li_2_C_2_O_4_@KB composite.

### 2.2. Electrode Preparation

The cathode slurry was prepared by thoroughly mixing NCM622 (Canrd, Dongguan, China), Li_2_C_2_O_4_@KB, Super P (Cuike, Shanghai, China)., and PVDF (Arkema, Shanghai, China) in a mass ratio of 72:8:10:10. This homogeneous mixture was then coated on the aluminum foil (Canrd, Dongguan, China) for half-cell and pressed into cathodes with a diameter of approximately 10 mm for full-call, designated as NCM622@LCKB electrodes. The areal density of the electrodes was approximately 7.5–8.5 mg·cm^−2^. Employing an identical experimental protocol, NCM622 control electrodes were fabricated by mixing NCM622, Super P, and PVDF in a mass ratio of 8:1:1. The resulting electrode sheets exhibited an areal density ranging from 7.5 to 8.5 mg·cm^−2^. Lithium foil was used as the counter electrode, Celgard 2500 was used as the separator, NCM622/NCM622@LCKB was used as the positive electrode to fabricate the half-cells. For the anode, a graphite-based slurry was formulated using graphite (active material), Super P (conductive agent), and PVDF (binder) in a mass ratio of 91.6:1.8:6.6. This slurry was uniformly coated onto a copper foil (Canrd, Dongguan, China) current collector and dried at 100 °C for 12 h. The dried electrode was then punched into discs with a diameter of 10 mm, achieving a mass loading density of approximately 2.1 mg·cm^−2^ with respect to the graphite active material. All electrodes were further dried at 80 °C for 2 h in a vacuum oven prior to cell assembly to remove residual moisture.

### 2.3. Materials Characterization

The morphology and microstructure of the Li_2_C_2_O_4_@KB composite and the resulting electrodes were examined using scanning electron microscopy (SEM, JSM-7100F, JOEL, Akishima, Tokyo) and transmission electron microscopy (TEM, Themis G2 300, FEI, Hillsboro, OR, USA). Elemental composition and distribution were analyzed by energy-dispersive X-ray spectroscopy (EDS) coupled with the SEM system. The crystalline structure of the samples, including the Li_2_C_2_O_4_@KB composite electrode before and after cycling, was characterized by X-ray diffraction (XRD, D8 Discover, Bruker, Billerica, MA, USA). Specific surface area and pore size distribution were measured via nitrogen adsorption using a BET surface area analyzer (ASAP 2020, Micromeritics, Norcross, GA, USA). Chemical bonding and functional groups were further investigated by Fourier-transform infrared spectroscopy (FT-IR, Nexus, Thermo Nicolet, Madison, WI, USA) and Raman spectroscopy (LabRAM HR, HORIBA, Paris, France). Thermogravimetric (TG, TG 209 F1, Netzsch, Bavaria, Germany) was used to characterize the Li_2_C_2_O_4_@KB composite and the degree of thermal decomposition of Li_2_C_2_O_4_. X-ray photoelectron spectroscopy (XPS, Kratos AXIS Supra, Shimadzu, Shimadzu, Japan) was used to characterize the surface elemental composition of the Li_2_C_2_O_4_@KB composite electrode after cycling. Prior to XPS analysis, the cycled working electrode was rinsed with dimethyl carbonate (DMC) to eliminate residual electrolyte and subsequently dried overnight under an argon atmosphere within the glovebox.

### 2.4. Electrochemical Characterization

The entire battery assembly procedure was carried out inside an argon-filled glovebox, where both oxygen and moisture levels were consistently maintained below 0.5 ppm. Lithium foil (Canrd, Dongguan, China) Celgard 2500 (Celgard, Charlotte, NC, USA), and NCM622/NCM622@LCKB were used as the counter electrode, separator, and positive electrode, respectively, to fabricate the half-cells. Each cell was filled with 80 μL of electrolyte, which contained 1 M LiPF6 dissolved in a mixture of ethylene carbonate (EC) and dimethyl carbonate (DMC) with a volume ratio of 3:7, was injected into each half-cell. Full cells with an N/P ratio of approximately 1.2 were constructed using NCM622/NCM622@LCKB as the positive electrode material and graphite (Gr) as the negative electrode material; Copper foil was used as the current collector for the negative electrode. All electrochemical evaluations were performed at 25 °C. Galvanostatic cycling tests were carried out using a Land battery test system. Cyclic voltammetry (CV) and electrochemical impedance spectroscopy (EIS) measurements were conducted with an electrochemical workstation (Solartron Analytical, EC-Lab VMP3, Bio-Logic, Grenoble, French).

## 3. Results

**Figure 2:**
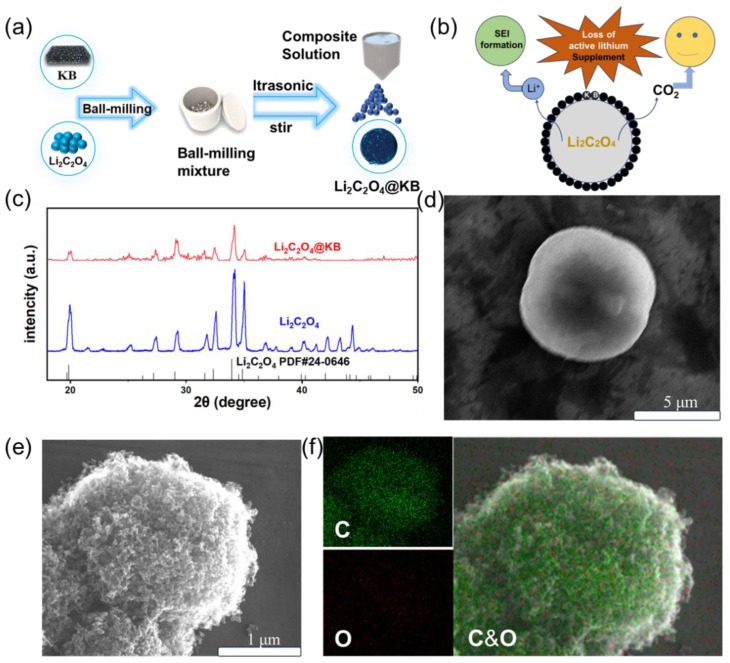
(**a**) Schematic illustration of the synthesis process of Li_2_C_2_O_4_@KB composite. (**b**) Mechanistic diagram of the lithium replenishing process of Li_2_C_2_O_4_@KB. (**c**) XRD profiles of Li_2_C_2_O_4_@KB and Li_2_C_2_O_4_. (**d**) SEM images of Li_2_C_2_O_4_ (**e**) SEM images of Li_2_C_2_O_4_@KB. (**f**) EDS-mapping of Li_2_C_2_O_4_@KB.

Figure 2a presents a schematic diagram outlining the synthesis procedure of the Li_2_C_2_O_4_@KB composite. The synthesis process commenced by reducing commercially available, irregularly shaped Li_2_C_2_O_4_ particles to the nanoscale through a straightforward and efficient high-energy ball-milling step. Subsequently, to enhance the electrochemical performance and reduce the decomposition voltage of the active material, the nanoscale Li_2_C_2_O_4_ is uniformly mixed with KB through ultrasonication in pure water, followed by spray-drying to form the final Li_2_C_2_O_4_@KB composite nanoparticles. Figure 2b depicts the lithium supplementation mechanism of the Li_2_C_2_O_4_@KB composite. During the initial charging process, Li_2_C_2_O_4_ decomposes electrochemically, releasing active lithium ions and generating gaseous carbon dioxide (CO_2_) as a by-product. The released CO_2_, being an inert gas, does not participate in any subsequent electrochemical reactions that would consume active mass. As a result, the additive does not adversely affect the reversible capacity of the cell. These active lithium ions migrate toward the anode, effectively compensating for the irreversible lithium loss associated with SEI formation. This process significantly enhances the Coulombic efficiency and improves the overall electrochemical performance of the lithium-ion battery.

Figure 2c presents the X-ray diffraction (XRD) patterns of the as-prepared Li_2_C_2_O_4_@KB composite, pristine Li_2_C_2_O_4_, and the standard PDF card (24-0646) for Li_2_C_2_O_4_. All characteristic diffraction peaks of the Li_2_C_2_O_4_@KB nanocomposite precisely match those of the standard reference and the pristine material. This result clearly indicates that the crystal structure of Li_2_C_2_O_4_ remains intact and unaltered after the ball-milling and compositing processes. The morphological evolution of the materials was characterized by scanning electron microscopy (SEM). Figure 2d displays the images of the raw commercial Li_2_C_2_O_4_ particles, revealing their large size. In contrast, the Li_2_C_2_O_4_@KB composite nanoparticles (shown in Figure 2e) exhibit a significantly carbon layer surface and uniform morphology after the ball-milling and compositing process. Figure S1 shows large-scale area of commercial Li_2_C_2_O_4_ particles and Li_2_C_2_O_4_@KB composite nanoparticle. The elemental distribution and core–shell architecture of the composite were further verified by energy-dispersive X-ray spectroscopy (EDS) mapping. As shown in Figure 2f, The uniform distribution of the carbon (C) signal, originating from the Ketjen Black (KB) coating, combined with the concentrated oxygen (O) signal from the Li_2_C_2_O_4_ core, offers clear evidence for the successful formation of a Li_2_C_2_O_4_@KB core–shell structure. Figure S2 shows EDS-mapping of commercial Li_2_C_2_O_4._

**Figure 3:**
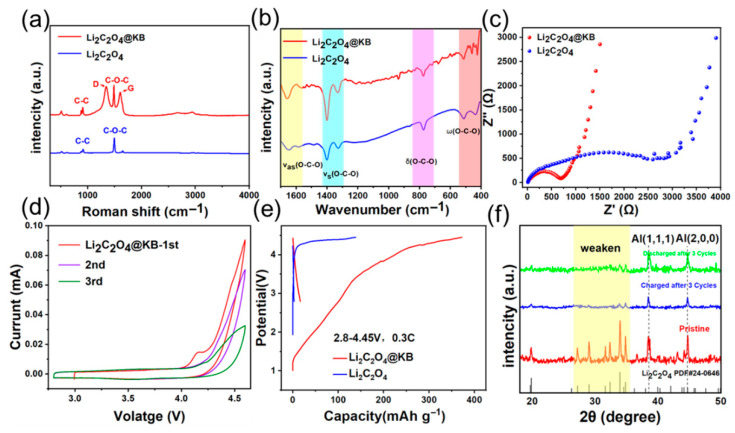
(**a**) The Raman spectrum curves for Li_2_C_2_O_4_ and Li_2_C_2_O_4_@KB. (**b**) FT-IR spectra of Li_2_C_2_O_4_ and Li_2_C_2_O_4_@KB. (**c**) EIS plots of Li_2_C_2_O_4_ and Li_2_C_2_O_4_@KB. (**d**) The CV profile of the Li_2_C_2_O_4_@KB half-cells for the first three cycles (scan rate: 0.1 mV s^−1^). (**e**) Initial charge–discharge curves for Li_2_C_2_O_4_ and Li_2_C_2_O_4_@KB half-cells at 2.8 to 4.45 V at 0.3 C. (**f**) XRD profiles of Li_2_C_2_O_4_@KB nanocomposite electrode before cycling, after three cycles in the charged state, and after three cycles in the discharged state.

Figure 3a presents the Raman spectra of Li_2_C_2_O_4_ @KB and pristine Li_2_C_2_O_4_, clearly revealing the composite’s structural characteristics. The spectrum of Li_2_C_2_O_4_@KB shows characteristic double peaks at 1350 cm^−1^ (D band, representing sp^3^ carbon defect vibrations) and 1580 cm^−1^ (G band, corresponding to sp^2^ carbon in-plane stretching vibrations), confirming the successful incorporation of KB carbon. Simultaneously, both spectra exhibit sharp peaks at 1492 cm^−1^, attributed to the symmetric stretching vibration of C-O-C bonds in Li_2_C_2_O_4_, which serves as a characteristic fingerprint of the Li_2_C_2_O_4_ phase. Additional vibrational modes at 920 cm^−1^ (symmetric C-C stretching) and 540 cm^−1^ (out-of-plane C-O-C rocking) are well-preserved in the composite spectrum, demonstrating that the crystalline structure of Li_2_C_2_O_4_ remains intact throughout the nanocomposite formation process. The coexistence of these distinctive spectral features provides compelling evidence for the successful formation of the Li_2_C_2_O_4_ @KB composite without compromising the structural integrity of either component.

Figure 3b displays the Fourier transform infrared (FTIR) spectroscopy results, which further corroborate the chemical bonding states in the Li_2_C_2_O_4_@KB composite. The spectrum shows characteristic vibrational modes of Li_2_C_2_O_4_: the peak at 1654 cm^−1^ (within the 1560–1660 cm^−1^ range) corresponds to asymmetric C-O-C stretching vibrations; the peaks at 1323 cm^−1^ and 1400 cm^−1^ (within 1320–1420 cm^−1^) represent symmetric C-O-C stretching vibrations; the peak at 774 cm^−1^ (within 740–800 cm^−1^) arises from in-plane C-O-C bending vibrations; the peaks at 440 cm^−1^ and 510 cm^−1^ (within 420–520 cm^−1^) correspond to out-of-plane C-O-C bending vibrations. The preservation of all these characteristic vibrational modes in the composite spectrum indicates that the KB coating process does not significantly alter the intrinsic lattice vibration modes or chemical structure of Li_2_C_2_O_4_, confirming the successful formation of the composite without structural degradation. Figure S3 presents the Brunauer–Emmett–Teller (BET) surface area analysis results for the prepared materials. The Li_2_C_2_O_4_@KB nanocomposite exhibits a substantially enhanced specific surface area of 194.5 m^2^ g^−1^, representing an order-of-magnitude increase compared to the pristine Li_2_C_2_O_4_ (18.2 m^2^·g^−1^). This remarkable 10.7-fold improvement in surface area provides significantly more active sites for electrochemical reactions and facilitates improved electrolyte infiltration. The substantially enlarged interfacial contact area between the active material and electrolyte is expected to enhance charge transfer kinetics and promote more complete utilization of the Li_2_C_2_O_4_ phase, thereby contributing to superior electrochemical performance in lithium-ion battery applications.

Figure 3c presents the electrochemical impedance spectroscopy (EIS) results of Li_2_C_2_O_4_ and Li_2_C_2_O_4_@KB nanocomposite half-cells. The charge transfer resistance (Rct) of Li_2_C_2_O_4_@KB, derived from the semicircle in the Nyquist plot, is approximately 672 Ω, whereas that of pristine Li_2_C_2_O_4_ is about 3000 Ω. This notable reduction in Rct demonstrates that the KB coating accelerates interfacial charge transfer, thereby significantly boosting the electrochemical activity of the Li_2_C_2_O_4_@KB nanocomposite. Figure 3d displays the cyclic voltammetry (CV) profile of the Li_2_C_2_O_4_@KB composite measured at a scan rate of 0.1 mV s^−1^, while Figure S4 presents the first three consecutive CV cycles of the pristine Li_2_C_2_O_4_. The Li_2_C_2_O_4_@KB nanocomposite exhibits a distinct oxidation peak at 4.26 V during the first anodic scan, which corresponds to its decomposition and lithium release process. In contrast, pristine Li_2_C_2_O_4_ shows a similar but higher-potential oxidation peak at 4.38 V. The intensity of these oxidation peaks diminishes dramatically in the second and third cycles, indicating that the lithium compensation occurs primarily during the initial cycle. This electrochemical behavior confirms that the Li_2_C_2_O_4_@KB nanocomposite undergoes irreversible decomposition at a reduced potential of 4.26 V, which is 0.12 V lower than that of pristine Li_2_C_2_O_4_ (4.38 V). Figure 3e presents the initial charge–discharge profiles of Li_2_C_2_O_4_ and Li_2_C_2_O_4_@KB nanocomposite half-cells measured between 2.8–4.45 V at 0.3 C. The Li_2_C_2_O_4_@KB nanocomposite exhibits a distinct charge plateau at approximately 4.26 V with a specific charging capacity of 372.3 mAh·g^−1^, while showing a negligible discharge capacity of only 16.5 mAh.g^−1^. This pronounced asymmetry between charge and discharge capacities confirms the highly irreversible electrochemical decomposition mechanism of the nanocomposite. In contrast, pristine Li_2_C_2_O_4_ demonstrates a higher charge plateau around 4.38 V with a substantially lower charging capacity of 138.3 mAh·g^−1^. These comparative results clearly demonstrate that the KB-based nanocomposite design simultaneously achieves both a reduced decomposition potential (decreased by 0.12 V) and a significantly enhanced lithium-supply capacity (increased by 169.2%), highlighting its superior functionality as an efficient pre-lithiation additive for cathode compensation in lithium-ion batteries.

Figure S5 presents the voltage-capacity profiles of the Li_2_C_2_O_4_@KB nanocomposite over three consecutive cycles. The composite delivers a cumulative capacity of 475.9 mAh·g^−1^ during these three cycles, with the majority of capacity being irreversibly extracted during the first cycle (372.3 mAh·g^−1^), followed by significantly diminished capacity in subsequent cycles. This rapid capacity decay pattern further confirms the sacrificial nature of the Li_2_C_2_O_4_@KB composite as a pre-lithiation agent, designed to provide a single, large initial lithium supplement rather than reversible cycling capability. Figure S6 presents dQ/dV curves of Li_2_C_2_O_4_ and Li_2_C_2_O_4_@KB. The decomposition oxidation peak for Li_2_C_2_O_4_@KB is observed at 4.26 V, while that for Li_2_C_2_O_4_ is located at a higher potential of 4.38 V. Figure 3f presents the X-ray diffraction (XRD) patterns of the Li_2_C_2_O_4_@KB nanocomposite electrode before cycling, after three cycles at the charged state, and after three cycles at the discharged state. The characteristic diffraction peaks of pristine Li_2_C_2_O_4_ remain well-preserved in the initial nanocomposite electrode, with all major peaks maintaining perfect alignment with the standard Li_2_C_2_O_4_ pattern. This result confirms that the KB coating process does not modify the intrinsic crystalline structure of the Li_2_C_2_O active material. However, after three complete charge–discharge cycles, most characteristic Li_2_C_2_O_4_ peaks disappear completely in both charged and discharged states, with only the characteristic peaks derived from the current collector—aluminum foil remained. This transformation provides clear evidence that the Li_2_C_2_O_4_@KB nanocomposite undergoes complete decomposition during electrochemical cycling, thereby fulfilling its function as a sacrificial lithium source for pre-lithiation. The combined electrochemical data consistently demonstrate the effectiveness of KB modification in lowering the decomposition potential while maintaining high lithium donation capacity.

**Figure 4:**
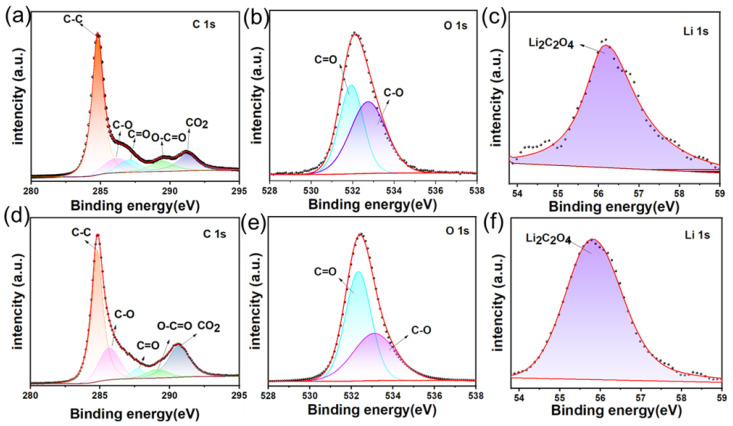
XPS spectra at initial states (**a**–**c**) and charged states after three cycles (**d**–**f**).

Figure S7 displays the full-range XPS spectra of the Li_2_C_2_O_4_@KB nanocomposite electrode under two different electrochemical states. The high-resolution C 1s spectrum (Figure 4a) of the pristine electrode exhibits five carbon species: C–C (284.0 eV, reference), C–O (286.1 eV), C=O (287.1 eV), O–C=O (289.4 eV), and a CO_2_-related peak (291.3 eV). The oxygen-containing functional groups (C–O, C=O, and O–C=O) originate from Li_2_C_2_O_4_, while the CO_2_ peak is associated with carbon black adsorption from air. This confirms the successful integration of Li_2_C_2_O_4_ and carbon components while preserving their chemical identities. After three cycles at the fully charged state (Figure 4d), the C 1s spectrum shows the same five components: C–C (284.0 eV), C–O (285.7 eV), C=O (287.7 eV), O–C=O (289.1 eV), and a CO_2_ peak at 290.6 eV. A notable increase in CO_2_ content is observed, attributed to the irreversible decomposition of Li_2_C_2_O_4_ during charging, which releases CO_2_ and active Li^+^ ions, confirming its role as a sacrificial pre-lithiation agent. The O 1s spectrum of the pristine electrode (Figure 4b) displays dominant peaks at 531.8 eV (C=O) and 532.8 eV (C–O). After cycling (Figure 4e), an increase in C=O content and a decrease in C–O bonds are observed, consistent with Li_2_C_2_O_4_ decomposition and CO_2_ release. The Li 1s spectrum of the pristine electrode (Figure 4c) shows a peak at 56.2 eV, characteristic of Li_2_C_2_O_4_. After cycling (Figure 4f), the peak shifts to 55.8 eV, indicating decomposition into lithium species. Quantitative analysis of functional groups in the C 1s spectra and lithium content from survey spectra (Figure S6 and Table 2) reveals higher CO_2_ content and lower Li ratios at the charged state. The content of CO_2_ changes from 35% to 44% and the ratios changes from 18.84% to 7.13%. These results confirm the decomposition of Li_2_C_2_O_4_ into electrochemically inert CO_2_, accompanied by the release of active lithium ions. In conclude, the XPS analysis across C 1s, O 1s, and Li 1s levels elucidates the electrochemical decomposition pathway of Li_2_C_2_O_4_@KB. The transformation from Li_2_C_2_O_4_ to Li^+^ and CO_2_, provides clear evidence of an irreversible sacrificial mechanism. These findings demonstrate the feasibility of Li_2_C_2_O_4_@KB as an efficient pre-lithiation agent for compensating irreversible capacity.

Despite its high theoretical specific capacity, the practical application of Li_2_C_2_O_4_ is limited by a low initial Coulombic efficiency, primarily attributable to its poor electronic conductivity and the irreversible decomposition of the oxalate anion. In contrast, KB is incorporated to mitigate these issues by providing a conductive network and a high specific surface area, which enhances electrolyte infiltration and electron transport, thereby improving the utilization of the active material. In this study, the mass ratio of Li_2_C_2_O_4_ to KB in the composite was maintained at 2:1. This composite was then incorporated into the cathode at a mass ratio of 10 wt%. Consequently, the actual mass fraction of KB from the composite within the entire cathode is only 3.33 wt%. This value is significantly lower than the 10 wt% of Super P that is conventionally added as the primary conductive agent, indicating that the KB in our composite primarily functions as a coating and reaction promoter rather than merely serving as a conductive additive. Consequently, the minimal incorporation of KB ensures that its impact on the overall energy density of the cathode is negligible. This advantage, combined with its effective lithium supplementation capability, positions the Li_2_C_2_O_4_@KB composite as a highly promising pre-lithiation additive for next-generation high-energy-density LIBs.

**Figure 5:**
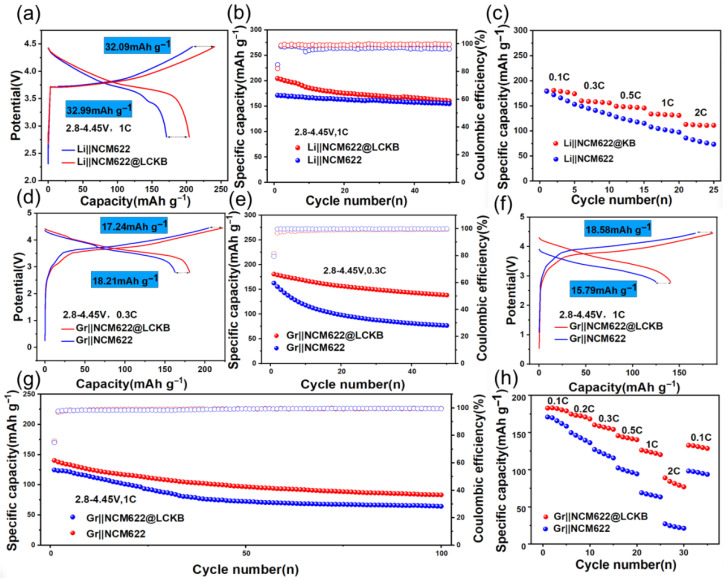
(**a**) Initial charge–discharge curves of the half-cells of Gr||NCM622 and Gr||NCM622@LCKB in the range from 2.8 to 4.45 V at 1 C. (**b**) Cycling performance of half-cells of Gr||NCM622 and Gr||NCM622@LCKB in the range from 2.8 to 4.45 V at 1 C. (**c**) Initial charge–discharge curves of the half-cells of Gr||NCM622 and Gr||NCM622@LCKB in the range from 2.8 to 4.45 V at 0.1 C, 0.2 C, 0.3 C, 0.5 C, 1 C, and 2 C. (**d**) Initial charge–discharge curves of the full cells of Gr||NCM622 and Gr||NCM622@LCKB in the range from 2.8 to 4.45 V at 0.3 C and (**f**) at 1 C. (**e**) Cycling performance of full cells of Gr||NCM622 and Gr||NCM622@LCKB in the range from 2.8 to 4.45 V at 0.3 C and (**g**) at 1 C. (**h**) Gr||NCM622 in the range from 2.8 to 4.45 V at 0.1 C, 0.2 C, 0.3 C, 0.5 C, 1 C, and 2 C.

To quantitatively evaluate its efficacy, the Li_2_C_2_O_4_@KB composite was added to NCM622 cathodes, and its impact on half-cell electrochemical performance was systematically investigated. Figure 5a presents the initial charge–discharge profiles of the NCM622 and NCM622@LCKB half-cells, measured between 2.8 and 4.45 V at a rate of 1 C. The NCM622@LCKB cathode demonstrates a significantly enhanced initial charging capacity of 240.7 mAh·g^−1^, which is 32.09 mAh·g^−1^ higher than that of the pristine NCM622. This substantial increase provides direct evidence of successful lithium compensation from the Li_2_C_2_O_4_@KB additive. The introduction of the Li_2_C_2_O_4_@KB additive incorporates additional conductive carbon into the electrode, thereby enhancing its overall electronic conductivity. This improvement contributes to the increase in discharge capacity by 32.99 mAh·g^−1^. Figure 5b compares the cycling stability of the cathode over 50 cycles under the same voltage window and current rate, further illustrating the positive impact of the pre-lithiation additive on long-term electrochemical performance. The high released capacity indicates extensive lithium insertion and extraction processes occurring within the material’s crystal structure [37,38,39,40]. These repeated volumetric changes induce gradual accumulation of micro-scale strain, ultimately leading to progressive degradation of the lattice and overall structural deterioration. As a result, the specific capacity declines significantly after 50 cycles, eventually approaching that of the unmodified NCM622 cathode. The 50-turn capacity retention of Li||NCM622@LCKB is 78.35% and the Li||NCM622 is 90.36%. Li||NCM622 and Li||NCM622@LCKB cells demonstrate well-synchronized capacity profiles during rate capability testing (Figure 5c), confirming the excellent kinetic performance of the cathode. The Li||NCM622@LCKB cell demonstrates significantly enhanced rate capability compared to the Li||NCM622 cell. For the Li||NCM622@LCKB half cells, the discharge capacity is 178.75, 157.97, 147.52, 132.34, 111.24 mAh·g^−1^ at 0.1, 0.3, 0.5, 1, 2 C, respectively. For the Li||NCM622 half cells, the discharge capacity is 165.7, 140.8, 120.9, 102.1, 78.8 mAh·g^−1^ at 0.1, 0.3, 0.5, 1, 2 C, respectively. Figure S8 shows the capacity-voltage profiles of the NCM622@LCKB and the NCM622 half cells It can be seen that the rate capacity of NCM622@LCKB half cells, especially the high rate, is much better than that of NCM622 half cells. Based on the promising electrochemical performance observed in half-cells, Li_2_C_2_O_4_@KB is confirmed to release a considerable amount of active lithium ions, thereby significantly enhancing the capacity of the NCM622||LCKB half-cell. To further comprehensively evaluate the improvement in electrochemical performance afforded by this pre-lithiation agent, subsequent studies were conducted by incorporating the material into a full cell configuration.

Full cells were assembled with a graphite anode and either NCM622 or NCM622@LCKB cathodes (designated Gr||NCM622 and Gr||NCM622@LCKB, respectively). Their electrochemical characterization evaluated the practical efficacy of the Li_2_C_2_O_4_@KB composite as a pre-lithiation additive in a realistic configuration. Figure S9 presents the initial charge–discharge voltage profile of the graphite anode, which exhibits a specific capacity of 374.05 mAh·g^−1^ at a rate of 0.1 C. The irreversible capacity loss of 21.29 mAh·g^−1^ in the first cycle is primarily attributed to the consumption of lithium ions during the formation of the SEI. Figure 5d compares the charge–discharge capacities of Gr||NCM622 and Gr||NCM622@LCKB full cells cycled between 2.8–4.45 V at 0.3 C. The Gr||NCM622 cell delivers initial charge and discharge capacities of 204.69 mAh·g^−1^ and 162.43 mAh·g^−1^, respectively. In contrast, the Gr||NCM622@LCKB cell shows significantly enhanced capacities of 221.93 mAh·g^−1^ (charge) and 180.8 mAh·g^−1^ (discharge), representing improvements of 17.24 mAh·g^−1^ and 18.21 mAh·g^−1^, respectively. These results clearly demonstrate the effective lithium replenishment provided by the Li_2_C_2_O_4_@KB additive in the cathode.

Figure 5e compares the cycling performance of Gr||NCM622 and Gr||NCM622@LCKB full cells over 50 cycles at 0.3 C within the voltage window of 2.8–4.45 V. The Gr||NCM622@LCKB cell demonstrates significantly enhanced capacity retention, maintaining 76.44% of its initial capacity after 50 cycles compared to only 47.22% for the Gr||NCM622 cell. As further illustrated in Figure S10, the capacity enhancement enabled by the Li_2_C_2_O_4_@KB additive is maintained over extended cycling, showing an 11.2% improvement in the initial cycle that increases progressively to 80.03% by the 50th cycle. This trend underscores the composite’s outstanding long-term stability and effective lithium compensation capability. Figure 5f presents the initial charge–discharge performance of Gr||NCM622 and Gr||NCM622@LCKB full cells at 1 C rate within the 2.8–4.45 V window. The Gr||NCM622 cell exhibits initial charge and discharge capacities of 166.8 mAh·g^−1^ and 124.73 mAh·g^−1^, respectively. In comparison, the Gr||NCM622@LCKB cell demonstrates significantly improved capacities of 185.38 mAh·g^−1^ (charge) and 166.8 mAh·g^−1^ (discharge), corresponding to substantial enhancements of 18.58 mAh·g^−1^ and 15.79 mAh·g^−1^, respectively. These improvements clearly indicate the effective lithium compensation provided by the Li_2_C_2_O_4_@KB additive, which contributes to reduced irreversible capacity loss in the initial cycle.

Figure 5g compares the 100-cycle performance of Gr||NCM622 and Gr||NCM622@LCKB full cells cycled at 1 C between 2.8–4.45 V. The Gr||NCM622@LCKB cell demonstrates superior capacity retention, maintaining 59.33% of its initial capacity after 100 cycles compared to 51.61% for the compare Gr||NCM622 cell. This 7.72% improvement in capacity retention highlights the positive impact of the Li_2_C_2_O4@KB additive in enhancing the long-term cycling stability of the full cell system. Figure S11 further illustrates the progressive capacity enhancement achieved by the Li_2_C_2_O_4_@KB additive across 100 cycles. The Gr||NCM622@LCKB full cells exhibit an immediate 12.66% capacity increase in the first cycle, with this improvement growing substantially to 29.52% by the 100th cycle, demonstrating the additive’s dual functionality in providing both initial lithium compensation and long-term cycling stability. Figure 5h presents the rate capability of Gr||NCM622 and Gr||NCM622@LCKB full cells at progressively increasing current densities from 0.1 C to 2 C. However, the Gr||NCM622@LCKB cells exhibit significantly improved rate capability compared to the Gr||NCM622 cells. Specifically, the discharge capacities of the Gr||NCM622@LCKB cells measure 182.11, 172.36, 157.12, 142.79, 123.48, and 81.43 mAh·g^−1^ at rates of 0.1, 0.2, 0.3, 0.5, 1, and 2 C, respectively. In contrast, the Gr||NCM622 cells display markedly lower capacities of 165.73, 142.88, 121.21, 97.99, 66.06, and 23.3 mAh·g^−1^ under identical test conditions. Notably, the enhancement becomes more pronounced at higher current rates, indicating the particular effectiveness of the Li_2_C_2_O_4_@KB additive in maintaining structural stability and facilitating lithium-ion transport under high-rate conditions. Figure S12 shows the capacity-voltage profiles of the NCM622@LCKB and the NCM622 full cells. These results demonstrate that Li_2_C_2_O_4_@KB is a highly effective lithium-supplementing additive for NCM622-based full cells. The incorporation of Li_2_C_2_O_4_@KB leads to significant improvements in both capacity and cycling performance, particularly in the Gr||NCM622 system, where the voltage range (2.8–4.45 V) is more conducive to the decomposition of Li_2_C_2_O_4_@KB. This confirms that Li_2_C_2_O_4_@KB is a viable and promising additive for enhancing the performance of LIBs.

## 4. Conclusions

In conclusion, this work proposes a novel strategy utilizing a well-designed Li_2_C_2_O_4_@KB core–shell nanocomposite as an efficient cathode pre-lithiation additive to significantly enhance the electrochemical performance of LIBs. The Li_2_C_2_O_4_@KB composite effectively reduces the decomposition potential to 4.26 V, compared to the 4.38 V plateau of pristine Li_2_C_2_O_4_. The practical feasibility of this approach is systematically validated in both Gr||NCM622 and Gr||NCM622@LCKB cells. In half-cell configurations, the additive increases the initial specific capacity by 32.99 mAh·g^−1^ at 1 C. More importantly, in full cells, it delivers an initial capacity improvement of 18.21 mAh·g^−1^ along with a 29.22% enhancement in capacity retention after 50 cycles at 0.3 C; at 1 C, the initial capacity increases by 15.79 mAh·g^−1^ with a 7.72% improvement in retention after 100 cycles. Rate performance tests reveal that the Gr||NCM622@LCKB cells exhibit discharge capacities have increased by 13.05, 17.17, 26.62, 30.24, and 32.44 mAh g^−1^, respectively, at 0.1, 0.2, 0.3, 0.5, 1, and 2 C. This study provides a simple and practicable methodology for developing high-energy-density LIBs through rational design of advanced cathode pre-lithiation agents.

## Figures and Tables

**Table 1:** Theoretical specific capacities of various lithium supplementation agents.

Variety	Theoretical Capacity
Li_2_C_2_O_4_	525 mAh g^−1^
Li_5_FeO_4_	684 mAh g^−1^
Li_2_NiO_2_	522 mAh g^−1^
Li_6_CoO_4_	489 mAh g^−1^
Li_2_S	1166 mAh g^−1^
Co/Li_2_O	~500 mAh g^−1^
Co/LiF	~500 mAh g^−1^

**Table 2:** Functional groups in the C spectra and the Li element content in the full spectra.

	C-O	C=O	O-C=O	CO_2_	Li
before cycles states	26%	19%	20%	35%	18.84%
charged states after three cycles	38%	8%	10%	44%	7.13%

## Data Availability

The original contributions presented in this study are included in the article/Supplementary Materials. Further inquiries can be directed to the corresponding author.

## Supplementary Materials

The following supporting information can be downloaded at: https://www.mdpi.com/article/10.3390/ma18194467/s1, Figure S1: Large-scale area SEM of Li_2_C_2_O_4_@KB and Li_2_C_2_O_4_; Figure S2: EDS-mapping of Li_2_C_2_O_4_; Figure S3: BET of Li_2_C_2_O_4_@KB (a) and Li_2_C_2_O_4_ (b); Figure S4: The CV profile of the Li_2_C_2_O_4_ half-cells for the first three cycles (scan rate: 0.1 mV S^−1^); Figure S5: Three cycles of charge/discharge curves of Li_2_C_2_O_4_ @KB at 2.8 to 4.45 V at 0.3 C; Figure S6: dQ/dV curves of Li_2_C_2_O_4_@KB and Li_2_C_2_O_4_; Figure S7: XPS spectra of the Li_2_C_2_O_4_@KB nanocomposite electrode at before cycles states (a) and at charged states after three cycles (b); Figure S8: Charge−discharge curves of the half-cells of (a) Gr||NCM622@LCKB and (b) Gr||NCM622 in the range from 2.8 to 4.45 V at 0.1 C, 0.3 C,0.5 C,1 C,2 C; Figure S9: Initial charge−discharge curves of the half-cells of graphite in the range from 0.005 to 2.0 V at 0.1 C; Figure S10: Discharge capacity at the 1st, 10th, 25th and 50th cycles of Gr||NCM622 and Gr||NCM622@LCKB in the range from 2.8 to 4.45 V at 0.3 C; Figure S11: Discharge capacity at the 1st, 25th, 50th, 75th and 100th cycles of Gr||NCM622 and Gr||NCM622@LCKB in the range from 2.8 to 4.45 V at 1C; Figure S12: Charge−discharge curves of the full-cells of (a) Gr||NCM622@LCKB and (b) Gr||NCM622 in the range from 2.8 to 4.45 V at 0.1 C, 0.2 C, 0.3 C, 0.5 C, 1 C, 2 C.
