# Supplementary material for: Promising Pre-Lithiation Agent Li2C2O4@KB for High-Performance NCM622 Cell
Source: Materials (Basel). 2025 Sep 25;18(19):4467. doi: 10.3390/ma18194467 (PMC12524966; doi:10.3390/ma18194467)
Supplement: Supplementary file 1 [file materials-18-04467-s001.zip › materials-3871016-supplementary.pdf]

materials

Article

Promising Pre-Lithiation Agent-$Li_2C_2O_4$@KB for High-Performance NCM622 Cell

Boqun Xia [1,2], Guangwan Zhang [1,2], Feng Tao [1,2] and Meng Huang [1,2,*]

[1] Sanya Science and Education Innovation Park, Wuhan University of Technology, Sanya 572000, China; 13407158120@163.com (B.X.); guangwanzhang@whut.edu.cn (G.Z.); t18438610309@163.com (F.T.)
[2] State Key Laboratory of Advanced Technology for Materials Synthesis and Processing, School of Materials Science and Engineering, Wuhan University of Technology, Wuhan 430070, China
* Correspondence: 211808@whut.edu.cn or 18717114932@163.com

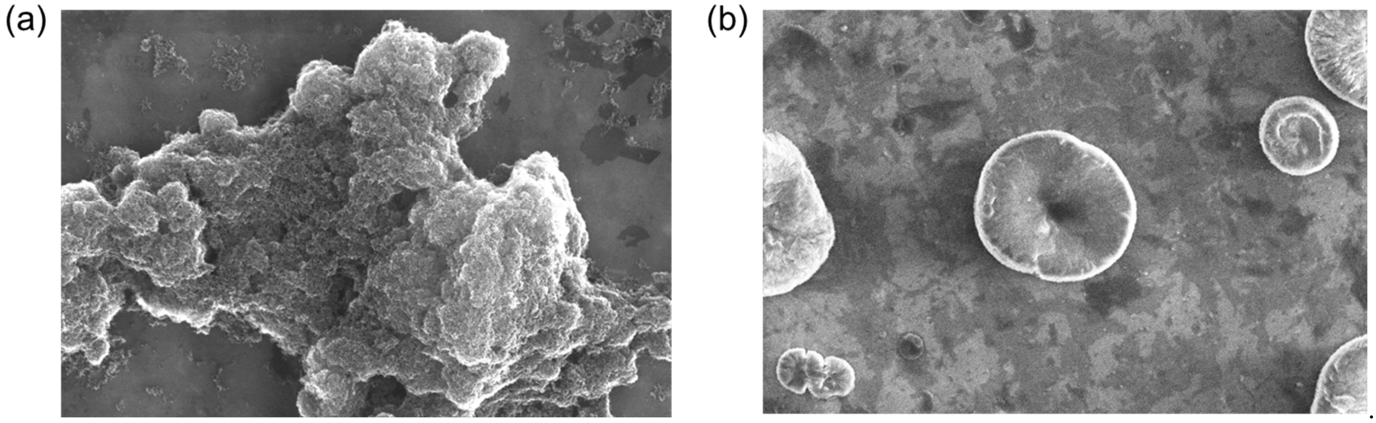

Figure S1. Large-scale area SEM of $Li_2C_2O_4$@KB and $Li_2C_2O_4$.

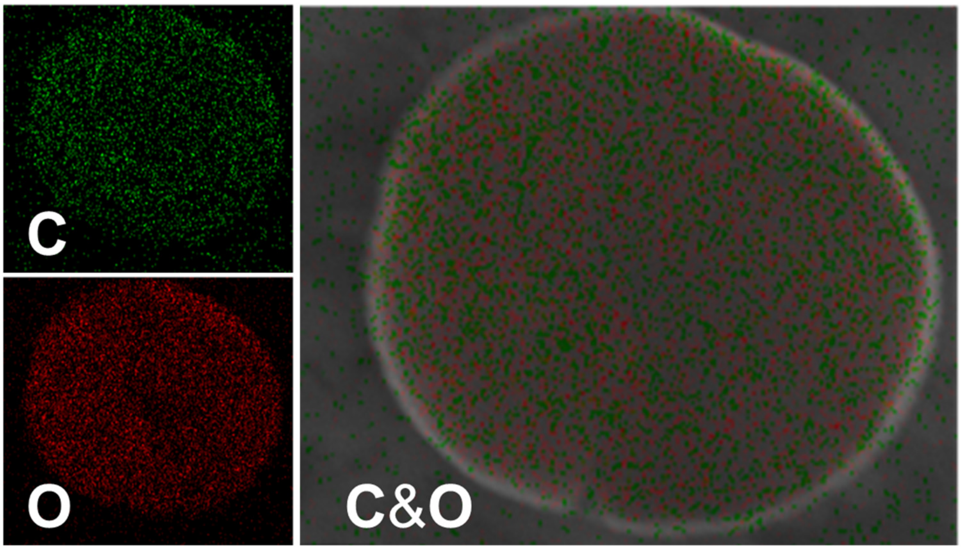

Figure S2. EDS-mapping of $Li_2C_2O_4$.

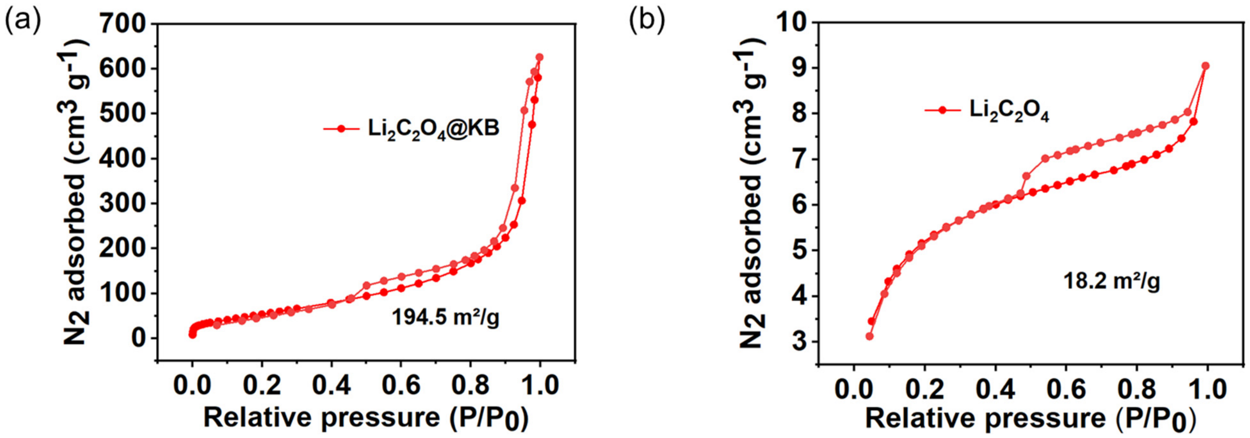

Figure S3. BET of $Li_2C_2O_4$@KB (**a**) and $Li_2C_2O_4$ (**b**).

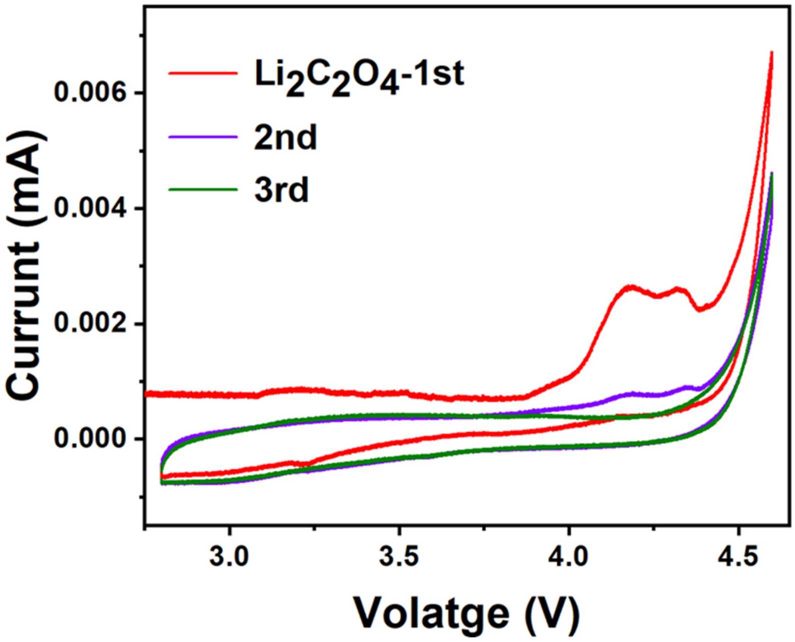

Figure S4. The CV profile of the $Li_2C_2O_4$ half-cells for the first three cycles (scan rate: 0.1 mV S-1).

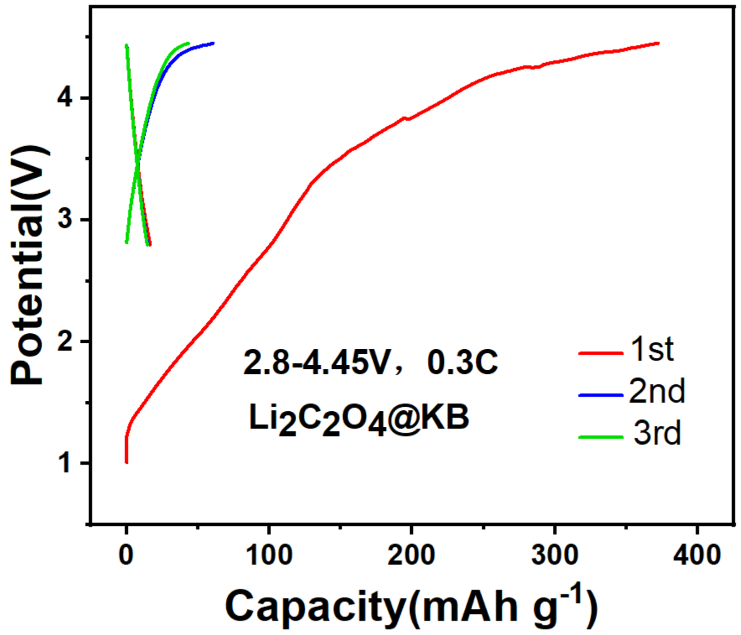

Figure S5. Three cycles of charge/discharge curves of $Li_2C_2O_4$@KB at 2.8 to 4.45 V at 0.3 C.

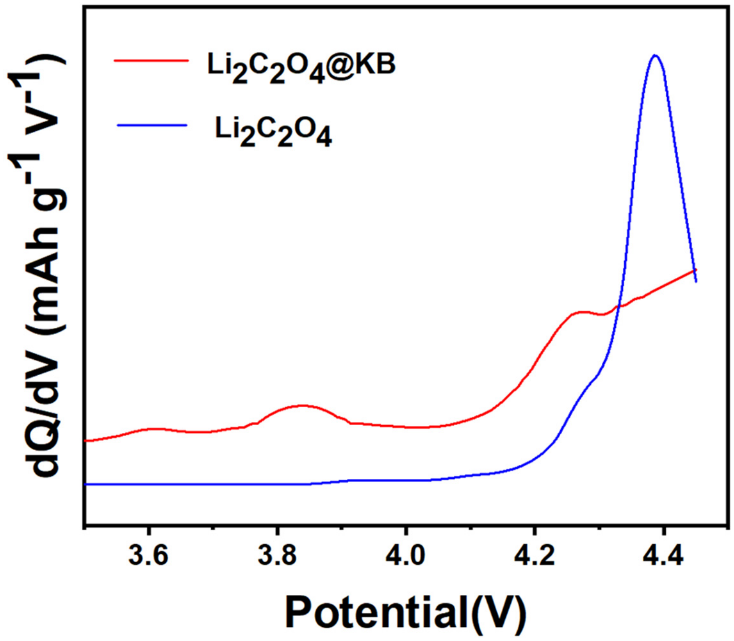

Figure S6. dQ/dV curves of $Li_2C_2O_4$@KB and $Li_2C_2O_4$.

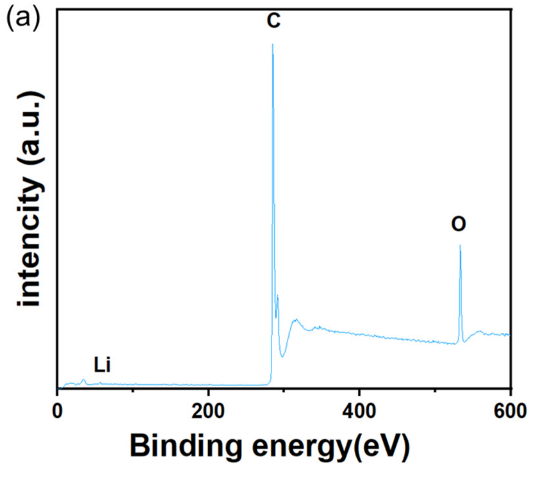

Figure S7. XPS spectra of the $Li_2C_2O_4$@KB nanocomposite electrode at before cycles states (**a**) and at charged states after three cycles (**b**).

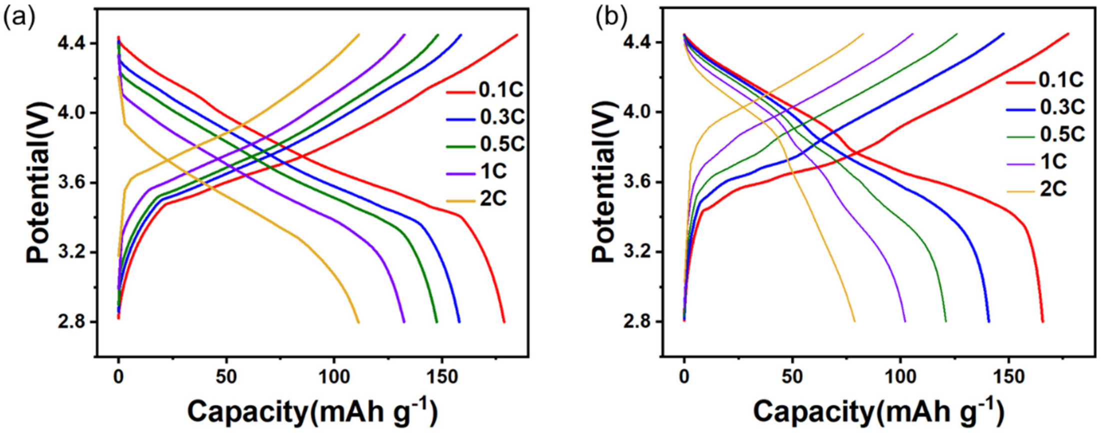

Figure S8. charge−discharge curves of the half-cells of (**a**) Gr||NCM622@LCKB and (**b**) Gr||NCM622 in the range from 2.8 to 4.45V at 0.1C, 0.3C, 0.5C, 1C, 2C.

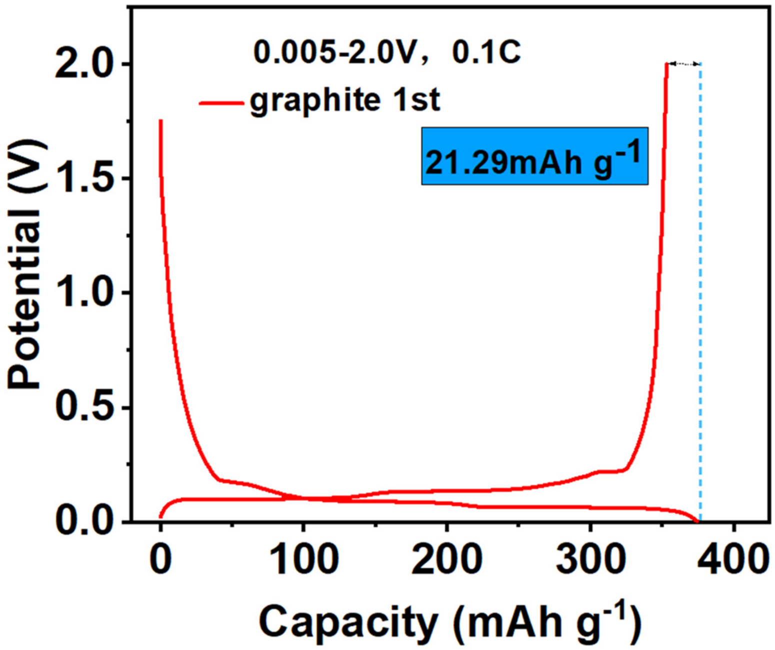

Figure S9. Initial charge−discharge curves of the half-cells of graphite in the range from 0.005 to 2.0V at 0.1C.

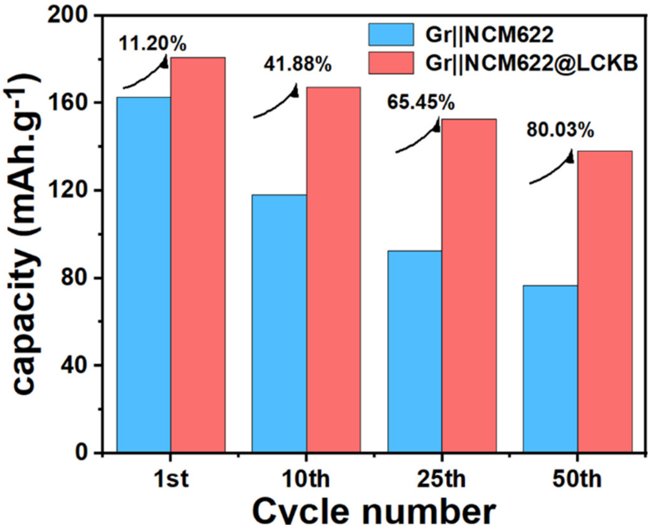

Figure S10. Discharge capacity at the 1st, 10th, 25th and 50th cycles of Gr||NCM622 and Gr||NCM622@LCKB in the range from 2.8 to 4.45V at 0.3C.

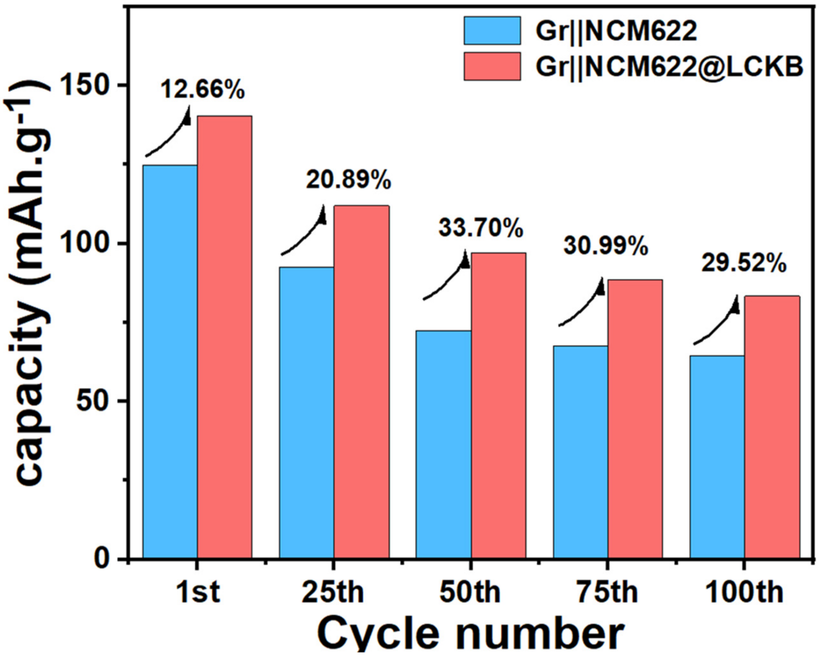

Figure S11. Discharge capacity at the 1st, 25th, 50th, 75th and 100th cycles of Gr||NCM622 and Gr||NCM622@LCKB in the range from 2.8 to 4.45V at 1C.

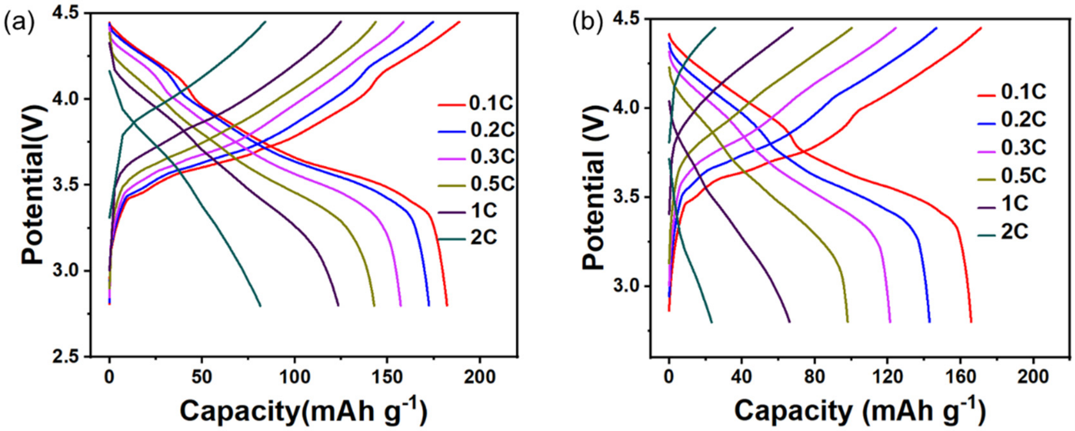

Figure S12. charge−discharge curves of the full-cells of (**a**) Gr||NCM622@LCKB and (**b**) Gr||NCM622 in the range from 2.8 to 4..45V at 0.1C, 0.2C, 0.3C, 0.5C, 1C, 2C.
